# Institutional capacity to generate and use evidence in LMICs: current state and opportunities for HPSR

**DOI:** 10.1186/s12961-017-0261-1

**Published:** 2017-11-09

**Authors:** Zubin Cyrus Shroff, Dena Javadi, Lucy Gilson, Rockie Kang, Abdul Ghaffar

**Affiliations:** 1 Alliance for Health Policy and Systems Research, World Health Organization, Avenue Appia 20, Geneva, 1211 Switzerland; 2 Health Economics Unit, Health Policy and Systems Division, School of Public Health and Family Medicine, University of Cape Town, Cape Town, South Africa; 3 Department of Global Health and Development, London School of Hygiene and Tropical Medicine, London, United Kingdom; 4 University of Queensland, Brisbane, Australia

**Keywords:** Health policy and systems research, Low- and middle-income countries, Institutional capacity, Alliance for Health Policy and Systems Research

## Abstract

**Background:**

Evidence-informed decision-making for health is far from the norm, particularly in many low- and middle-income countries (LMICs). Health policy and systems research (HPSR) has an important role in providing the context-sensitive and -relevant evidence that is needed. However, there remain significant challenges both on the supply side, in terms of capacity for generation of policy-relevant knowledge such as HPSR, and on the demand side in terms of the demand for and use of evidence for policy decisions. This paper brings together elements from both sides to analyse institutional capacity for the generation of HPSR and the use of evidence (including HPSR) more broadly in LMICs.

**Methods:**

The paper uses literature review methods and two survey instruments (directed at research institutions and Ministries of Health, respectively) to explore the types of institutional support required to enhance the generation and use of evidence.

**Results:**

Findings from the survey of research institutions identified the absence of core funding, the lack of definitional clarity and academic incentive structures for HPSR as significant constraints. On the other hand, the survey of Ministries of Health identified a lack of locally relevant evidence, poor presentation of research findings and low institutional prioritisation of evidence use as significant constraints to evidence uptake. In contrast, improved communication between researchers and decision-makers and increased availability of relevant evidence were identified as facilitators of evidence uptake.

**Conclusion:**

The findings make a case for institutional arrangements in research that provide support for career development, collaboration and cross-learning for researchers, as well as the setting up of institutional arrangements and processes to incentivise the use of evidence among Ministries of Health and other decision-making institutions. The paper ends with a series of recommendations to build institutional capacity in HPSR through engaging multiple stakeholders in identifying and maintaining incentive structures, improving research (including HPSR) training, and developing stronger tools for synthesising non-traditional forms of local, policy-relevant evidence such as grey literature. Addressing challenges on both the supply and demand side can build institutional capacity in the research and policy worlds and support the enhanced uptake of high quality evidence in policy decisions.

## Background

Despite the role of evidence in informing effective policy- and decision-making in health and optimising the use of scarce resources [1], in many countries, particularly low- and middle-income countries (LMICs), evidence-informed decision-making remains the exception rather than the rule. A paucity of evidence that is context sensitive, timely and relevant for decision-makers, challenges in accessing existent evidence and issues of capacity to appraise and use evidence at both individual and organisational levels within Ministries of Health (MoHs) are all important reasons for this [2]. The lack of relevant, context sensitive and timely research evidence to inform decision-making can be significantly explained by the traditional separation of research generation from policy- and decision-making processes. This is also not helped by academic incentive structures that prioritise publication in high-impact journals over policy relevance of research as the main metric for career advancement. Access to research evidence is hampered by (1) its presentation, usually in the form of peer-reviewed journal articles, and (2) physical accessibility due to journal paywalls that impede access for many LMIC-based policy- and decision-makers [2, 3].

Where evidence is available, capacity to appraise and use different kinds of evidence remains weak. This is both at the level of individual decision-makers who may not have the time or incentives to interpret evidence, as well as at the level of MoHs, which in contrast to many high-income countries (HICs), may not have defined processes to consider and use evidence at different stages of the decision-making process [2]. The overall result of this is a vicious cycle of low demand for evidence to inform policies, its inadequate generation, and its low utilisation in policy- and decision-making. Policy adoption, design and implementation are thus often sub-optimal, resulting in health systems failures and lack of response to population needs [2].

Health policy and systems research (HPSR)^1^ has a crucial role in addressing this situation, a role demonstrated in Mexico’s *Seguro Popular* health insurance scheme and Thailand’s national Universal Health Coverage programme; two prominent examples where locally generated HPSR evidence informed the design and implementation of programmes and policies to strengthen health systems [2, 4–6]. There are several reasons for this. First, at an epistemological level, HPSR goes beyond the more positivist paradigms of biomedical research, embracing critical realist and relativist perspectives, which allows for an understanding of evidence that “*is defined with respect to specific decision-making contexts*”, as opposed to one that is “*unconstrained by context*” [1, 7]. It also enables a move away from ‘evidence hierarchies’ that judge the quality of decision-making almost solely in terms of narrowly conceptualised ‘evidence quality’, with randomised controlled trials serving as a gold standard, to an orientation that prioritises evidence relevance and applicability, attributes that have been identified to play a significant role in whether evidence makes its way into decision-making [1, 7].

Second, HPSR evidence sheds light on issues including what health systems are and what needs to be done to strengthen them to improve health, and how to influence policy agendas to take up activities to strengthen health systems, design them and implement them, from the various disciplinary perspectives that make up the field, including economics, sociology, public health and political science [7]. Reflecting this holistic understanding, HPSR evidence has a broad remit, and can relate to the macro-level or the wider context in which policies are made [8], the meso-level or the institutional arrangements and processes within which policies are designed and implemented [9], as well as the micro-level or how individuals impact policy change [7, 10], and can use either quantitative [9], qualitative [8] or mixed methods [10].

Third, recognising that evidence that is relevant for policy- and decision-making goes well beyond research published in peer-reviewed journals, HPSR evidence includes programme evaluation reports, routine data including that generated through the Health Management Information System (HMIS), as well as the more intangible but experiential ‘tacit’ knowledge that decision-makers widely use in their day to day activities, but only rarely systematically codify for wider application [7].

Fourth, HPSR prioritises policy relevance of research over rigid methodological and disciplinary boundaries and emphasises the role of policy-makers, programme managers and implementers in informing research agendas, including through engagement with researchers during the research process as the major consumers of research products [7].

As more light is shed on the value of HPSR in strengthening health systems, financial resources for the field have increased and a gradual increase in production of HPSR publications can be seen over the past decade [11, 12]. In spite of this, significant challenges remain in institutional capacity for HPSR generation and the uptake of HPSR and research evidence more widely.

### Institutional capacity for HPSR

The generation of high-quality research needs more than just skilled researchers, just as its incorporation into decision-making goes beyond individual champion decision-makers [13]. Individuals need support in the form of organised and well-functioning institutions with appropriate and well-aligned institutional arrangements to generate and use evidence to inform decision-making processes.

Experience from many countries, including Mexico and Thailand, demonstrates that strong, well-governed and well-functioning research institutions such as FUNSALUD (Mexican Foundation for Health) and the International Health Policy Programme, have played a significant role in the generation and dissemination of HPSR, leading to major changes in health policy at the national level [14]. This role has been greatly catalysed by also having in place institutional capacity within national MoHs to appraise, synthesise and use evidence to inform policy- and decision-making. In spite of this, the central role of developing in-country health systems research institutions in efforts to strengthen health systems remains inadequately recognised [13]. Instead, funders have tended to prioritise efforts to develop skills at the level of individual researchers [2].

Recognising both the strong inter-linkages between knowledge generation and utilisation and the role of relevant institutions in these areas in enabling the evidence-to-policy process, we bring together, in one paper, our analysis of institutional capacity^2^ to generate HPSR and to use evidence to inform decision-making in LMICs. We do this through surveying major research institutions engaged in HPSR as well as MoHs from across the world. We go on to suggest measures that could be taken by relevant stakeholders to strengthen institutions engaged in evidence-to-policy processes and address the gaps identified. Our work emphasises organisational and system level arrangements for HPSR (including policies, rules and incentives) rather than an analysis of institutions’ physical infrastructure and human resources, as this is the focus of previous work in this area [15–17]. Second, with respect to MoHs, it goes beyond issues of policy-maker training and interactions with researchers to identify (the existence or lack thereof) organisation and system level incentives for MoHs to demand and use research evidence and strategies to further develop these.

## Methods

### Literature review

A desk review of the literature was carried out pertaining to two thematic areas, namely (1) capacity of research institutions to generate HPSR and (2) incorporation of research evidence into decision-making for health, including the capacity of decision-makers to use research evidence. This was achieved through an online search using the Google Scholar search engine, complemented by an examination of reference lists of initial articles identified, as well as the authors pre-existing knowledge of key literature in this area.

While there exists a substantial body of literature that examines the enablers and barriers to the incorporation of research evidence into decision-making processes and how to overcome them, the literature on capacity of institutions to generate and use research evidence in decision-making for health is less developed. In particular, we were unable to find published literature that could shed light on processes established at the level of MoHs to facilitate the uptake and use of research evidence for decision-making at a cross-national level.

Research institution capacity for HPSR has been explored through global level surveys by Gonzalez-Block and Mills [15], Bennett et al. [16] and Adam et al. [17]. Additional research in this area has been performed by Bennett et al. [18], examining factors enabling the development of six health policy research institutions across Africa and Asia. This is complemented by a regional level analysis by Simba et al. [19] examining research institutions in East and Central Africa, and by Mirzoev et al. [20], who assessed capacity for HPSR in seven African universities across five countries associated with the CHEPSAA (Consortium for Health Policy and Systems Analysis in Africa) project. Finally, country-specific assessments of institutional capacity have also been explored through CHEPSAA in South Africa, Ghana and Nigeria [21–23].

Enablers and barriers to evidence incorporation in decision-making have been examined both in HICs [24–28], and increasingly in LMICs [29–31]. The more specific literature on policy-maker capacity to use research has largely focused on strengthening individual level capacities through training programmes and engagement with policymakers. Examples of this include the work of Pappaioanou et al. [32], who examine the use of a training strategy in four LMICs to familiarise decision-makers with using data and evidence to inform their work, and the paper by Jauregui et al. [33], which looks at lessons learnt on strengthening technical capacity for evidence-informed decision-making for new vaccines as part of PAHOs ProVac initiative. The key findings from the literature review are provided in Box 1.

Box 1 Key findings from the literature review

**Table:** 

Institutional Capacity to Generate HPSR
**•** Challenges of funding – low total funding; unsteady funding and over-reliance on international sources, implications for sustainability of HPSR research institutions
**•** Human resource challenges – lack of critical mass of HPSR researchers; HPSR researchers scattered across institutions; difficulty in retaining HPSR researchers when competing with international organisations/consultancy firms
Enablers and barriers to evidence-informed decision-making
**•** Enablers
**○** early engagement of decision-makers in research process
**○** creating awareness among decision-makers of available research
**○** trust between researchers and decision-makers
**○** research perceived as topical and timely by decision-makers
**○** research dissemination in formats appropriate for decision-makers
**°** providing technical skills to decision-makers in interpreting evidence, including through on-going training
**•** Barriers
**○** lack of technical capacity among decision-makers to interpret evidence
**○** research dissemination in formats difficult to read and interpret
**○** research that is not seen as timely or relevant

### Data sources

Data for this paper was obtained through two email-administered survey questionnaires. The first survey, focusing on knowledge generation processes for HPSR, was targeted at research institutions engaged in HPSR relevant to LMICs. The second, a survey of MoHs, aimed to understand the capacities within MoHs in LMICs to demand and use evidence for the purposes of improving policy- and decision-making.

The survey on knowledge generation processes was administered between July and December 2014. An invitation email was sent to 481 research institutions, including universities, independent research institutions, think tanks and international organizations. India (n = 40), Nigeria (n = 26) and China (n = 24) were the countries where the highest number of invitations were sent. The institutions included partners and grantees of the Alliance for Health Policy and Systems Research (henceforth the Alliance)^3^ as well as other institutions identified on the basis of representation at the 2012 Second Global Symposium on Health Systems Research. Institutions conducting HPSR relevant to LMICs were included, irrespective of whether they were located in LMICs. For Alliance partners and grantees, the email was sent to the email address of the individual listed in the Alliance database. For institutions identified on the basis of representation at the Global Symposium on Health Systems Research, one researcher within each institution was sent the email. However, individuals (typically senior researchers, programme directors) were asked to respond for the department/institution as a whole. Contacts were provided with a writable pdf file in which they were asked to fill their responses. A total of six reminder emails were sent to follow-up with respondents. A total of 110 responses were received, corresponding to a response rate of 23%. India and China, with 14 and 7 institutions, respectively, were the countries with the highest number of institutions among the responders.

The survey instrument contained questions pertaining to definitional issues around HPSR, institutional arrangements to facilitate HPSR, incentives provided to individual researchers to undertake HPSR, linkages with decision-makers, as well as questions around constraints facing the field and priority areas for future research. World Bank geographical regions and income groups were used to classify countries. Income classifications are as per World Bank criteria released in July 2015.

A total of 39 MoHs were targeted for the purpose of the second survey, performed in the first half of 2015. Care was taken to ensure that the sample had adequate geographic spread while ensuring that the MoHs of the largest LMICs, i.e. China, India, Indonesia, Brazil, Pakistan, Bangladesh and Nigeria, were included in the sample. The survey included questions on sources of research evidence for MoHs and barriers to evidence use, practices in using evidence, and policy and legislative mechanisms to incentivize use of evidence. Overall, 24 responses were received, a response rate of nearly 62%.

For both surveys, data were initially entered in Excel. Survey data were analysed using Stata 13 software to generate tables of descriptive statistics. Both survey questionnaires (which were designed to complement previous work in this area as discussed in the earlier section on institutional arrangements for HPSR) were developed after intensive discussions within the Alliance Secretariat. Draft questionnaires were commented on by leading researchers and policy-makers represented on the Alliance’s Scientific and Technical Advisory Committee with questionnaires being revised in response to comments received. In terms of overlaps between the two surveys, both surveys contained questions to understand mechanisms for researcher decision-maker engagement, which though complementary, do not allow for the results to be directly comparable.

## Results and Discussion

### Survey of research institutions

#### Background

The 110 institutions were based in 56 countries. Sub-Saharan Africa accounted for 25% of responses, the most for any region; conversely, institutions in the Middle East and North Africa region accounted for only 4% of the responses received. Overall, 15% of the institutions were based in low-income countries (LICs); HICs accounted for 23% of the institutions. Nearly 63% of the institutions were based in middle-income countries (MICs). Nine institutions reported that they had not conducted any HPSR study during the 5 years prior to the survey and were thus not asked any further questions. All results henceforth pertain to the remaining 101 institutions. Key findings from the research institution survey are summarized in Box 2.

#### Defining the field

In spite of the rapid growth of HPSR and the crystallisation of a scientific community in this area, only 35% (*n* = 101) of institutions reported that their institution had a shared definition of HPSR that was known and understood by all researchers.

Among institutions noting a shared definition, HPSR was most commonly defined in terms of research related to the six building blocks of the health system. Alternative definitions included “*a multidisciplinary research field focusing on development and implementation of local and global health policies, system strengthening, services and promotion, and influence of key stakeholders on their outcomes*” and “*an emerging trans-disciplinary global field with its own evolving standards for creating, evaluating, and utilizing knowledge, and distinguished by a particular orientation towards influencing policy and wider action to strengthen health systems.*”

#### Core funding is far from the norm, especially in LICs

A little over one-third (34%) of institutions in the sample reported receiving any core research funds (defined as funds not tied to an individual research project) (n = 99). While 54% of HIC institutions (n = 24) received some core funding, only 31% of institutions in MICs (n = 65) and 10% of institutions in LICs (n = 10) received any core funds. In a majority (54%) of institutions, core funds accounted for less than 25% of total research funding. HIC institutions received a higher proportion of their total funds from core funding as compared to those in LMICs.

Box 2 Key findings of the research institution survey

**Table:** 

• Lack of a shared definition of HPSR: 35% of research institutions reported having a definition for HPSR
• Low prevalence of core funding, particularly in LICs: 34% of research institutions received any core funding for HPSR (54% in HICs, 31% in MICs and 10% in LICs)
• Incentive structures for policy-relevant research remain under-developed: Publication continues to be most important promotion criteria (48% of respondents)
• Funding and inadequate numbers of trained researchers are major constraints to HPSR production: human resource problem particularly important in LMICs
• Leadership and governance identified by most respondents as a topical area where more research is needed
• Researcher and decision-maker linkages are largely informal, formal linkages such as Memoranda of Understanding were reported by less than half of respondents

#### Academic incentive structures for HPSR remain underdeveloped

The further development of the HPSR research community is contingent on attracting young researchers to commit themselves to the field. This is particularly challenging as the products of HPSR are not always suitable for publication in high-impact journals [13, 14]. Alternative incentive structures are thus needed for HPSR researchers.

Publication record was ranked as the most important criteria for promotion by 48% of respondents, whereas 26% of respondents ranked the ability of research to impact policy as the single most important promotion criteria, a positive finding for an applied field like HPSR (*n* = 92).

In total, 36% of institutions reported having put in place incentives for individuals to carry out policy-relevant research (n = 100). However, only two institutions reported the creation of separate career tracks for policy-relevant research such as “Professor of Practice”, with career advancement not as directly linked to publication in high-impact journals as regular tenure track positions.

#### Funding and trained human resources are the most cited constraints

Research funding was cited as the most serious constraint facing HPSR knowledge production by 57% of respondents, followed by human resource constraints (25%); 11% of respondents opined that issues around the nature of HPSR (including lack of disciplinary homogeneity, definitional issues and questions of rigor), were the most serious constraints to HPSR knowledge generation. However, respondents from LMICs were far more likely to rank human resource constraints as the most important constraint (31%) than those from HIC-based institutions (8%).

#### More research is needed on leadership and governance

Respondents were asked to identify areas within HPSR where they believed there were the most significant gaps in the literature and where research was most needed. Given the widespread use of WHO’s six-building blocks framework to describe and understand health systems, respondents were asked to identify areas in terms of these building blocks. Leadership and governance was identified by nearly half of all respondents (49%) as the area where most research was needed; this was followed by health service delivery (17%) and health financing (12%) (n = 90). These rankings remained largely consistent across country income groups.

#### Researcher decision-maker linkages, though common, are largely informal

Respondents were asked whether their institutions had formal or informal linkages with National or State level MoHs or public health bodies that aimed to produce research to inform policy design and implementation. Formal linkages were specified as Memoranda of Understanding or Commissioned Research, while personal interactions were classified as informal linkages.

The presence of formal or informal linkages was reported by 93% of respondents (n = 101). However, only 46% reported that there was a formal linkage in place that brought researchers and decision-makers together to identify relevant research areas. The conversion of research findings into recommendations that could be used by policy-makers was mandatory (in the form of a written rule or administrative requirement) in only 30% of research institutions

### Survey of MoHs

#### Background

To complement survey results from research institutions, MoHs were surveyed, providing an understanding of the mechanisms in place that influence how evidence is or is not used. Table 1 provides data on the number of countries in each region responding to this survey. Respondents were most often based either in the Office of the Director General of Health (25%) or in the Planning and Policy Unit of the MoH (25%); 62.50% of them had received a doctoral or professional degree. Females accounted for 9 of the 24 respondents (37.50%). Key findings from the survey of MoHs are provided in Box 3.

**Table 1:** Regional breakdown of responses received from Ministries of Health (MoHs)

	MoHs invited	Responses received
Africa	11	6
East Asia and Pacific	10	7
Europe and Central Asia	5	3
Latin America and Caribbean	5	3
Middle East and North Africa	3	1
South Asia	5	4
	39	24

Box 3 Key findings – Ministries of Health (MoH) survey

**Table:** 

• Health management information systems and Ministry internal reports are the most important sources of evidence for decision-making: 45% and 15% of respondents ranked these as the most important sources
• Unavailability of locally relevant evidence and poor presentation of evidence are the main barriers to obtaining evidence for decision-making
• Making research available to MoH staff is not prioritised: only 54% of MoHs systematically collate evaluations and unpublished data
• High self-reported use of research but weak mechanisms and incentives to enable this: 79% of MoHs report using research evidence to inform decision-making, but only 42% have specific arrangements with research institutions to support commissioning of research
• Training in accessing and using research often provided to individuals within MoHs, but longer term arrangements (sabbaticals, secondments, rotations) to expose decision-makers to research institutions are uncommon

#### HMIS and Ministry internal reports are main sources of evidence

**Fig. 1:**
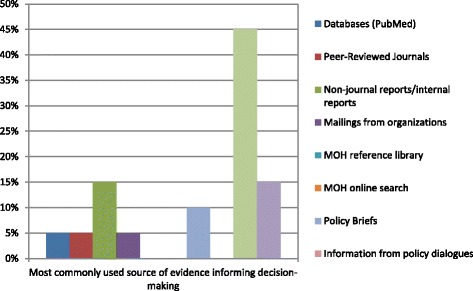
Most common sources of evidence for informing decision-making

Routine HMIS data and Ministry internal reports were the most often used sources of evidence for 45% and 15% of respondents, respectively (Fig. 1). This demonstrates the need for researchers to actively engage with MoHs on an ongoing basis to understand their research needs, develop questions together and communicate research findings through easily accessible media beyond peer-reviewed publications, including policy briefs and dialogues, which can both serve as sources of evidence and inform Ministry internal reports.

#### MoHs face several barriers to obtaining relevant evidence

The two most cited barriers to obtaining relevant evidence for decision-making were reported to be the unavailability of locally relevant applied research (30%) and poor presentation of research findings, making it difficult for policy-makers to understand them (30%). The next most cited reason was inadequate communication between researchers and decision-makers about policy-relevant research (25%).

#### Making research available to staff is not prioritised

There appears to be inadequate attention to bringing together and enabling the use of existent research evidence such as that contained in internal reports that could inform and strengthen decision-making. Only a little over half (54%) of MoHs reported that they systematically collated evaluations, and other sources of unpublished data for staff to use to inform their work. Of these, a little over one third reported that they had put this collection online, demonstrating the relatively low priority given to making research evidence available to MoH staff.

#### High self-reported use of research but weak mechanisms and incentives to enable this

In most MoHs (79%), respondents reported having used research evidence to directly inform a policy decision in the year leading up to the survey. The same proportion of respondents (79%) affirmed that the MoH engaged with researchers during decision-making processes and that the MoH sponsored research to inform its decision-making.

However, there do not appear to be institutional mechanisms or incentives in place to facilitate this intention in practice. At the individual level, research use was found to serve as a performance indicator for any staff member in a little over 20% of MoHs; this includes staff in areas such as monitoring and programme evaluation. Additionally, less than one-third (29%) of MoHs reported having in place mechanisms, such as sabbaticals or secondments, that would enable their staff members to gain experience at research institutions. Similarly, at the level of the organisation, less than half (42%) of MoHs reported having formal Memoranda of Understanding with research institutions when commissioning research.

Similarly, while a majority of MoHs appear to recognise the importance of research appraisal and programme evaluation in informing policy decisions, the data suggest that these issues are approached in an arbitrary fashion in a majority of settings. Policies or legislative mechanisms mandating the evaluation of MoH programmes were reported by over 70% of MoHs. However, there was little clarity on what qualified as an evaluation, with less than a third of these Ministries reporting having in place guidelines laying down specific criteria for what constituted an acceptable evaluation of a programme.

#### Training decision-makers to demand and use evidence – a mixed picture

Putting in place legislative and policy measures and information-rich inventories to facilitate evidence-informed decision-making will amount to little in the absence of officials trained in accessing and using research evidence within the MoH. Continued education, imparted through ongoing training programmes, and mechanisms enabling the rotation of staff between the MoH and research institutions are two distinct strategies to facilitate the bringing together of the worlds of research and policy.

A little under half (11 of 23) of respondents, reported having received training relevant to accessing or using research evidence in decision-making processes in the two years prior to the survey. Skills frequently imparted included those in data analysis, carrying out general internet searches and skills to access databases such as PubMed. One respondent reported receiving training in the production and dissemination of evidence briefs for policy and yet another reported that the training received had been to “assess *the quality of research evidence,* [and in]*… methodologies, tools and resources in using evidence in policy-making*”.

Only seven MoHs reported having in place mechanisms to enable staff rotation to research institutions. Of these, secondment mechanisms were in place at three MoHs and one MoH reported allowing officials time for sabbaticals at research institutions. From the data, it would appear that, while research training programmes for MoH officials are not uncommon, mechanisms to enable more in-depth exposure to research institutions over a longer period of time are less prevalent. This is not surprising given the shortage of skilled human resources in a large number of MoHs particularly in LMICs.

Finally, respondents from MoHs were asked an open ended question to identify facilitators to the uptake of evidence in decision-making in their MoHs. In line with the pre-existing literature in this area, increased communication and collaboration between researchers and decision-makers, increased availability of relevant knowledge, and the timing of research coinciding with reforms were identified as leading facilitators.

## Recommendations and conclusion

Based on our findings, we suggest actions to take forward the generation of HPSR and the use of HPSR and evidence more broadly for informing health-related decision-making. These require concerted and coordinated efforts on the part of a variety of stakeholders, including funders and international agencies, national governments and the HPSR community.

The challenges inherent in developing a shared definition of HPSR and improving alignment in this field, even within institutions, are reflected in the low proportion of institutions reporting having such a definition. While definitions for HPSR have been developed, notably in the Alliance’s own products, including the Methodology Reader on HPSR [7], there remains a lack of common interpretation of the field across geographical and disciplinary boundaries, with negative consequences for how the field is perceived in terms of academic rigor. There is, therefore, a need to share these definitions and to harmonise the field as put forth in the 2011 series of seminal articles on Building the Field of HPSR [34–36]. Teaching and training materials on HPSR developed and disseminated by the CHEPSAA consortium have sought to do precisely this. The process of developing such a common understanding must be carried out through an open and transparent process to ensure inputs from the range of disciplines making up HPSR and dispel the fear of ‘disciplinary capture’ by positivist research traditions [36].

In addition, field building would also entail advancing research methods, developing common taxonomies and creating guidelines for the appropriate conduct and reporting of HPSR. This includes how best to judge the impact of complex interventions within dynamic and interconnected health systems, which randomised controlled trials that presume linear relationships between cause and effect fail to do [14]. While Health Systems Global, through its Thematic Working Groups, has an important role in this process at the global level, given the context specificity of HPSR, there is a specific need to spur the development of national research networks to encourage communication and field building at this level [35]. The establishment of new journals dedicated to HPSR is needed, especially at regional and national levels, to publish policy-relevant work that may be highly applicable and needed though not of interest to international audiences [35].

With many institutions involved in HPSR having their own networks of actors, improving harmonisation of the field and strengthening advocacy for its uptake in decision-making should be performed by bringing these networks together and developing a common mission and agenda for the way forward for HPSR, including through the identification of HPSR research priorities. The importance of this has been understood by the Alliance, which has introduced a new strategic objective in its Strategic Plan 2016–2020, centred around convening partners, especially policy-makers, to enable HPSR to better inform policy- and decision-making [37].

Funding is urgently needed to operationalise this. Funding for HPSR globally represents a figure that is merely 2% of the annual budget of the United States National Institutes for Health [11, 38]. We emphasise the need for concerted efforts to increase core funding for HPSR, particularly for institutions in LICs and lower-MICs. Core funding is important for a number of reasons; it allows for the establishment of institutional research infrastructure, it enables institutions to hire and retain research talent, which is in short-supply particularly in LICs, and finally it facilitates the ability of institutions to develop and work on their own research agendas in areas where project funding may not be available [2]. Without core funding, building national research capacity – a priority for many funding agencies – will continue to stagnate, making sustainability a challenge [2].

There is also need for multiple stakeholders, most importantly global and national HPSR funders and HPSR research institutions to come together to put in place incentives to encourage the generation of HPSR knowledge. Possible incentives include directing funding for the development of alternative career tracks, such as Professor of Practice, for researchers engaged in policy-relevant research that will prioritise policy relevance of an individual’s research as an indicator for career advancement. Developing metrics to measure the policy relevance of an individual’s research contribution and institutionalising the use of such metrics in research institutions is thus important [14]. This is specifically needed to attract and retain young researchers to work in HPSR, since a lot of HPSR, particularly that performed in the form of case studies, is not amenable to publication in high-impact journals, the chief metric for career advancement in academic institutions.

It is also important to do more to incentivise knowledge production beyond peer-reviewed publications and towards developing products of direct relevance to decision-makers, such as policy briefs, research summaries and the creative use of social media, if the HPSR knowledge produced is to have maximal impact in informing decision-making [3, 14]. This should be complemented by the creative use of new and emerging technologies, including geo-mapping, that can often provide decision-makers valuable information to inform their next course of action. There is an increasing recognition of the need to both generate and collate these knowledge products, as performed by networks including the Joint Learning Network on Universal Health Coverage and Communities of Practice such as those on results-based financing [39, 40].

The findings also bring to the fore the need for the greater production and availability of researchers trained in HPSR, with the difficulty in getting trained researchers being a particularly major challenge for research institutions in LMICs. This is evident from the finding that, though the production of HPSR about and in LMICs has increased, a substantial proportion of HPSR publications on LMICs are produced by authors from HICs. Even within LMICs, the production of HPSR is highly skewed, with a small number of countries accounting for a large share of HPSR publications [12].

Developing and offering more HPSR training programmes and funding for this is only part of the solution; it is equally important for HPSR institutions, particularly in LMICs, to attract talented researchers by putting in place clear career advancement and promotion avenues, including through mentorship programmes to support inter-disciplinary HPSR researchers who often work relatively isolated within departments focusing on particular disciplines [35]. Furthermore, creating supportive and attractive research environments can also sustain local talent. These would include access to publication databases and peer-reviewed literature, often a serious constraint for researchers based in LMIC research institutions.

The barriers faced by MoHs in obtaining research evidence demonstrate the need to increase access to research literature, both in terms of peer-reviewed publications as well as grey literature, including project reports, evaluations and other non-peer-reviewed materials. Encouraging open access publication (an area where there has been much growth in recent years) as well as enabling access to databases of peer-reviewed literature through institutional subsidies for decision-making bodies in LMICs are two potential mechanisms to facilitate access to peer-reviewed literature. It is worth noting that unintended consequences of some of these potential solutions should also be considered. For example, the higher fee of publication in open access journals could potentially create bias in the type and context of research being featured in these journals, as well as contributing to publication bias overall. This is an important consideration for funders and further stresses the need for core funding as a priority as opposed to project-based funds.

Collating and making available reports and evaluations (which are the most widely used sources of evidence for MoHs according to our findings above) is a more challenging task. There is an urgent need to develop repositories of this grey literature at provincial, national and global levels to ensure that this knowledge is systematically collated and brought together and to enable its potential use by relevant stakeholders to inform decision-making by placing it online. MoHs at the provincial and national levels and leading global health agencies all have a major role in setting up these repositories and making available this important global public health good [14].

To produce high quality reports and evaluations, it could be mandated that policy or programme documents be informed by a review of the existent and available literature and spell out how this evidence informed a given policy decision or justify why it was not used in the instances where this was the case. The development of institutions or agencies to evaluate public programmes, as has been done in a wide range of countries including Mexico, South Africa and Colombia, among others, is one potential way forward. National governments would do well to learn from the experiences of these and other countries of how systematic evaluation can improve the transparency and quality of decision-making.

Finally, these mechanisms need to be sustained through sensitisation of decision-makers in MoHs to debates on what constitutes evidence, the role of evidence in informing decision-making as well as imparting them with specific skills in accessing and interpreting evidence. This should be reinforced through the establishment of incentives to demand and use research at the individual and institutional levels. Examples of the former include making research usage a part of individual performance appraisal for decision-makers in relevant positions or units within MoHs, and putting in place arrangements to allow for rotations of MoH staff within research institutions to sensitise them to the potential role of research in informing decision-making [14].

There are some limitations of this paper. The representativeness of the sample both among research institutions and MoHs is one limitation that is inherent in the use of surveys to gather such information. Both research institutions and MoHs were sent multiple reminders to improve the response rates. The likelihood of questions being understood differently by different respondents was mitigated by pre-testing the survey instruments and making changes to enhance clarity. However, the lack of a common understanding of HPSR in itself could have had an effect on the lens through which questions were answered. Furthermore, there is the potential for results having been skewed due to a Hawthorne Effect, with respondents being aware of the promotion of use of evidence in decision-making by the Alliance and seeking to please the group conducting the survey by overemphasising the reality of evidence use while not reporting all challenges.

However, in spite of these limitations, we do believe that, by bringing together the state of knowledge generation and utilisation and bridging the perspectives of researchers and decision-makers, this paper has highlighted a number of major challenges to the generation of HPSR, and utilisation of research evidence more broadly, and demonstrated the need to understand both sides of the demand-and-use equation in order to establish sustainable mechanisms to overcome these challenges, enabling the co-production of HPSR knowledge by researchers and decision-makers [41]. In this vein, the paper offers specific, actionable solutions that take a multi-pronged approach by engaging the whole range of relevant stakeholders.

## Abbreviations

CHEPSAA: Consortium for Health Policy and Systems Analysis in Africa
HICs: high-income countries
HPSR: health policy and systems research
LICs: low-income countries
LMIC: low- and middle-income countries
MICs: middle-income countries
MoH: Ministry of Health.

## Footnotes

1 Health policy and systems research has been defined as “*a field that seeks to understand and improve how societies organize themselves in achieving health goals and how different actors interact in the policy and implementation processes to contribute to policy outcomes. By nature, it is inter-disciplinary, a blend of economics, sociology, anthropology, political science, public health and epidemiology that together draw a comprehensive picture of how health systems respond and adapt to health policies, and how health policies can shape and be shaped by health systems and the broader determinants of health*” [7].

2 For the purposes of this paper, we use UNDP’s definition of capacity as “*the ability of individuals, institutions, and societies to perform functions, solve problems and set and achieve objectives in a sustainable manner*” [42].

3 The Alliance for Health Policy and Systems Research is an international collaboration housed within WHO, Geneva, that seeks to promote the generation and use of health policy and systems research to strengthen health systems in low- and middle-income countries.
